# Packet Flow Capacity Autonomous Operation Based on Reinforcement Learning

**DOI:** 10.3390/s21248306

**Published:** 2021-12-12

**Authors:** Sima Barzegar, Marc Ruiz, Luis Velasco

**Affiliations:** Optical Communications Group, Universitat Politècnica de Catalunya, 08034 Barcelona, Spain; sima.barzegar@upc.edu (S.B.); marc.ruiz-ramirez@upc.edu (M.R.)

**Keywords:** reinforcement learning, autonomous network operation, offline/online learning

## Abstract

As the dynamicity of the traffic increases, the need for self-network operation becomes more evident. One of the solutions that might bring cost savings to network operators is the dynamic capacity management of large packet flows, especially in the context of packet over optical networks. Machine Learning, particularly Reinforcement Learning, seems to be an enabler for autonomicity as a result of its inherent capacity to learn from experience. However, precisely because of that, RL methods might not be able to provide the required performance (e.g., delay, packet loss, and capacity overprovisioning) when managing the capacity of packet flows, until they learn the optimal policy. In view of that, we propose a management lifecycle with three phases: (i) a self-tuned threshold-based approach operating just after the packet flow is set up and until enough data on the traffic characteristics are available; (ii) an RL operation based on models pre-trained with a generic traffic profile; and (iii) an RL operation with models trained for real traffic. Exhaustive simulation results confirm the poor performance of RL algorithms until the optimal policy is learnt and when traffic characteristics change over time, which prevents deploying such methods in operators’ networks. In contrast, the proposed lifecycle outperforms benchmarking approaches, achieving noticeable performance from the beginning of operation while showing robustness against traffic changes.

## 1. Introduction

Autonomous network operation evolves from software-defined networking (SDN) [1] and promises to reduce operational expenditures by implementing closed loops based on data analytics [2,3]. Such control loops can be put into practice based on policies that specify the action to be taken under some circumstance (policy-based management), e.g., allocate the capacity of a packet flow so that the ratio traffic volume over capacity is under 80%. In packet flows, this ratio is related to the average delay that the packets in the flow will experience because of queuing, and thus to the Service Level Agreement (SLA) in the case that the packet flow is related to some customer connection. Although such policies can be modified, they are purely reactive. Under high traffic variations, they might entail either poor Quality of Service (QoS) (e.g., high delay or even traffic loss) and SLA breaches or poor resource utilization, which in both cases represent large costs for network operators. Note that policy-based management does not define the desired performance and thus, agents implementing those policies are unable to learn the best actions to be taken. Another approach for network automation is Intent-Based Networking (IBN) [4,5]. IBN allows the definition of operational objectives that a network entity, e.g., a traffic flow, has to meet without specifying how to meet them. IBN implements and enforces those objectives, often with the help of Machine Learning (ML) [6]. Although many ML techniques could be potentially applied for the autonomous capacity operation of traffic flows, in this paper, we rely on Reinforcement Learning (RL) [7]. We consider several RL methods of different complexity and analyze their performance, in particular: (*i*) Q-learning; (*ii*) *dueling double deep Q-learning* (denoted D3QN) [8], and (*iii*) Twin Delayed Deep Deterministic Policy Gradient (TD3) [9].

With the deployment of network slicing [10] and the support to time-sensitive applications [11], the flow capacity autonomous operation (hereafter referred to as CRUX) focuses on answering a major problem that network operators are facing nowadays: how to allocate the right capacity to every traffic flow, so as to provide the desired QoS (e.g., by preventing traffic loss and ensuring a given maximum average delay), while minimizing overprovisioning (i.e., *capacity–traffic*).

The next subsections provide the needed background, review the related works, and present the main contributions of this work.

### 1.1. Background on RL methods

RL considers the paradigm of an *agent* interacting with its *environment* for learning reward-maximizing behavior (*learning agent*). At every discrete time step *t*, with a given state *s*, the agent selects action *a* with regard to a policy, and it receives from the environment a reward *r* and the new state *s’*. The objective is to find the optimal policy that maximizes the expected return.

The simplest RL is Q-learning [7], which is a model-free discrete RL method that is able to learn the optimal policy represented by a Q-table of pairs <*s*, *a*>, each containing a *q* value. Being at state *s*, an action *a* is taken—either the one corresponding to the highest *q* value, or chosen randomly. After the action is implemented, it is evaluated by receiving the new state, *s’,* and the gained reward *r* from the environment. The agent then updates the corresponding *q* value in the Q-table. Q-learning works efficiently for problems where both states and actions are discrete and finite. However, it suffers from two main problems: it introduces overestimation, which leads to suboptimal policies, and the Q-table grows with the number of states.

*Deep Q-learning* (DQN) substitutes the Q-table by a feed-forward deep neural network (DNN) that receives a continuous representation of the state and returns the expected *q* value for each discrete action [7]. Because the DNN tends to make learning unstable, countermeasures need to be taken. The most extended measure is to keep a *replay buffer* storing the last *experiences,* i.e., tuple <*s*, *s’*, *r*, *a*> that is used to retrain the DNN. In addition, *double DQN* [12] uses two different DNNs (*learning* and *target*) to avoid overestimation, which happens when a non-optimal action is quickly biased (due to noise or exploration) with a high *q* value that makes it preferably selected. Thus, the learning model is updated using the *q* values retrieved from the target DNN, which is just a simple copy of the learning model that is periodically updated. Finally, *dueling double DQN* (named D3QN in this paper) [8] uses two different estimators to compute the *q* value of a pair <*s*, *a*>: (*i*) the *value* estimator, which can be intuitively seen as the average *q* value of any action taken at state *s*; and (*ii*) the *advantage* estimator, which is the specific state action-dependent component. The sum of both value and advantage components returns expected *q* values.

DQN-based methods assume a finite discrete action space. If continuous state and action spaces are required, other approaches such as *Actor–Critic* methods [9] can be used. The main idea behind *Actor–Critic* methods is that two different types of models are trained separately: (*i*) *actors*, who are in charge of computing actions based on states, and (*ii) critics*, who are in charge of evaluating the actions taken by actors, i.e., to compute *q* values. Both actor and critic models can be implemented by means of DNNs. Among all different *Actor–Critic* methods, the Twin Delayed Deep Deterministic Policy Gradient (TD3) method [9] considers one single actor and two different critic models, where the minimum value from the two critics is used to learn, aiming at reducing overestimation.

### 1.2. Related Work

Several works have proposed the application of RL algorithms for autonomous network operation. In the context of optical networking, the authors in [13] proposed the use of *Actor–Critic*-based algorithms running in the SDN controller for dynamic lightpath provisioning. They showed that changes in the traffic profile impact the obtained performance. The authors of [14] extend their work from [13] to multi-domain scenarios where multiple RL agents improve their learning by transferring knowledge. The authors of [15] studied the application of several deep RL algorithms (including DQN) and reward functions to increase network availability in SDN-controlled wide area networks. In the context of IBN, the work in [16] applied Q-learning to the autonomous operation of optical connections based on subcarrier multiplexing. Their RL algorithm, running in the optical transponder, activated and deactivated subcarriers to adapt the capacity of the optical connection to the upper layer packet traffic. The authors of [17] proposed an IBN solution based on Q-learning, which demonstrated the ability to learn the optimal policy based on the specified operational objectives related to the allocated capacity or the delay. Finally, the work in [18] proposed an RL algorithm for autonomous bandwidth allocation in the context of multilayer optical networks. They proposed a centralized algorithm running in the SDN controller that supervises the network performance. When the network performance degrades, the centralized algorithm tunes parameters in the RL algorithms.

One of the key issues in the previous works is the time needed to learn optimal policies, as exploration entails low-reward decision making (i.e., far from optimal operation), as shown in [13]. In view of this, the performance of RL methods is typically evaluated after some training phase, i.e., when reward achieves a stationary behavior. However, a subject that is poorly or not even considered in the literature is when RL algorithms need to operate before they are properly trained. Note that this happens in our case, as the actual characteristics of the traffic flow are unknown until it is provisioned. Moreover, it is not realistic to assume, in general, the same conditions during training and operation phases, due to mid/long-term traffic evolution, which makes it difficult to reproduce highly accurate operation conditions during training. To solve these issues, the authors of [19] proposed a general learning lifecycle that included both offline training (e.g., in a sandbox domain [20]) and online learning, in the context of supervised ML. The objective of that work was to accelerate autonomous operation by deploying accurate models that are firstly trained offline and fine-tuned while in operation, thus adapting pre-trained models to actual in-field operation conditions.

### 1.3. Contributions

In this paper, we apply the main lessons learnt from the previous works to IBN agents based on RL. We assume that a packet flow (alternatively, referred as traffic flow or simply flow) conveying traffic with unknown characteristics is established and the allocated capacity needs to be set to ensure the required QoS (from the set-up time), while minimizing resource utilization. To this end, a policy-based management is used at the set-up time to start operating the capacity of the flow; meanwhile, traffic measurements are collected to characterize the traffic. Note that policy-based operation can be highly reliable, as it is based on specific rules that can be defined and understood by human operators. However, such an operation usually obtains poor resource utilization. Therefore, it would be useful to substitute policy-based operation by an RL model as soon as possible. To that end, pre-trained generally applicable models for the *partly* observed traffic characteristics are loaded and the RL algorithm starts operating. A per-flow algorithm supervises the performance of the RL algorithm and tunes model parameters to ensure the required QoS. Once enough traffic measurements are available, offline training is carried out in a sandbox domain to produce a specific model well-suited for that particular traffic flow, which then substitutes the generic one. Since the traffic characteristics can change over time, analysis must be continuously performed to detect them and change the operating model when needed. By iterating that cycle, the proposed RL agent will be able to adapt to traffic changes that would otherwise degrade performance.

The rest of the paper is organized as follows. The pros and cons of applying RL to real network operation are highlighted in Section 2, which include poor performance during the initial set-up and during changes in the traffic flow. The CRUX problem and the proposed approach are as follows: it targets operating a traffic flow and ensuring the required QoS from set-up time without previous knowledge of the traffic characteristics. The motivation behind the different phases of operation and the proposed lifecycle is presented, where pre-trained models and offline–online RL cycles aim at solving the identified issues. The CRUX problem is formally defined in Section 3. Several RL approaches can be used to solve CRUX, and each one requires different settings of states and actions. A methodological approach is provided to model the problem with different RL algorithms, and how the QoS targets are related to parameters in the reward function. Section 4 presents the algorithms that analyze the traffic characteristics, supervise the performance, and make decisions regarding the model and parameters to be used for operation. The discussion is supported by the results presented in Section 5. Finally, Section 6 concludes the paper.

## 2. The Flow Capacity Autonomous Operation (CRUX) Problem

### 2.1. Flow Capacity Autonomous Operation

**Figure 1:**
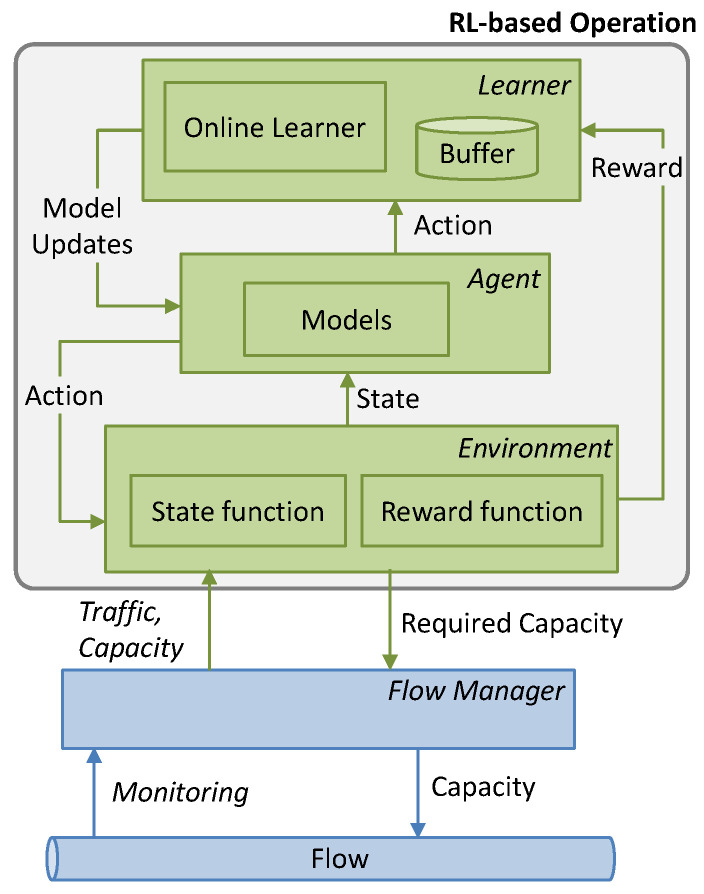
Flow capacity autonomous operation. RL framework with learner, agent, and environment.

Let us start by analyzing the result of the autonomous capacity operation of a traffic *flow*. A *flow manager* might collect monitoring data from the network and expose some interface, so an RL algorithm can take action. For illustrative purposes, Figure 1 shows a typical RL framework [21], where the learning agent is separated into two different blocks, the *learner* and the *agent*.

Let us assume that the monitoring data include the byte count since the last monitoring sample (amount of traffic) and the actual capacity allocated to the flow. The actions to be taken are related to the actual capacity allocated to the flow, which can be increased or decreased as needed with some *granularity* to meet the required QoS. For instance, a customer connection can manage the capacity of the flow with granularity 1 Gb/s by configuring some packet node, whereas in a virtual link supported by the optical layer, the capacity can be increased/decreased by establishing or tearing down parallel lightpaths, each with a capacity of 100 s Gb/s. It seems clear that the time to change the capacity is also different, ranging from seconds to minutes. The RL algorithm should then decide the capacity to be allocated to the flow to absorb *variation* in the traffic from one monitoring sample to the next, plus the time to increase the allocated capacity, with the objective to avoid any traffic loss and ensure some additional QoS metric. Then, the *traffic variation* becomes a major feature for a flow, together with the *traffic pattern*, i.e., the evolution of the mean traffic with time.

This approach can provide excellent performance once the policies that avoid traffic losses meet the desired QoS, and minimize overprovisioning are learned; however, online learning of such policies requires time. In addition, there are several issues that can impact the aforementioned online learning performance, e.g., (1) changes in traffic variability might produce loss before new policies are learned; (2) smooth model fine tuning could not be enough to mitigate persistent errors in taking some specific actions; and (3) online learning tends to forget valuable learning in the long run, thus reducing the model’s accuracy [7].

**Figure 2:**
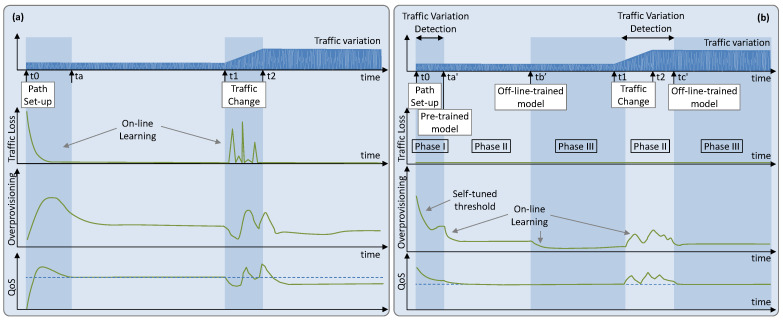
Operation lifecycle. (**a**) Online learning RL operation. (**b**) Offline training with online fine tuning RL operation.

Figure 2a represents a possible evolution of the traffic variation (the traffic pattern is omitted here for simplicity) and the obtained performance—overprovisioning, traffic loss, and some other QoS metric. The path supporting the flow is established at time *t_0_* and the desired QoS is specified, so the RL algorithm needs time to learn the traffic variation (and the traffic pattern); meanwhile (until *ta* in Figure 2a), poor performance, including traffic loss, can be expected. Once a good model is obtained, it is expected that an RL algorithm can provide the target performance. However, a steep change in the variation of the traffic (times *t*_1_ to *t*_2_) can impact the performance until the new variation is learned. Nonetheless, it might happen that the performance does not converge to the desire level even after learning the new traffic variation.

It seems clear that the above behavior is unacceptable for network operators, as it would provide poor performance and might incur penalties due to SLA breaches. Specifically, it seems of paramount importance to start the operation with already trained models. To that end, an initial model can be trained offline using a network simulator in a sandbox domain. Once in operation, the model will be improved by the online learner. However, there are traffic characteristics, e.g., traffic pattern, that are observed after a long period of time, e.g., several days. Therefore, some alternatives are needed to operate the flow during that initial time.

Our solutions go beyond training offline and propose implementing offline–online learning cycles to deal with large changes in traffic flow, i.e., to provide guaranteed performance during the whole lifetime of the traffic flow (Figure 2b). Specifically, (*i*) a policy-based management implemented as a self-tuned *threshold-based* algorithm is in charge of managing the flow capacity during the time immediately after the path is set up (*Phase I*: time interval [*t_0_*, *ta’*], where *ta’-t_0_* should be short, e.g., 1 h). That algorithm tunes a threshold for the flow capacity and accurately determines the traffic variation. This approach enables dynamic flow capacity allocation by fixing the right values for the threshold that minimize overprovisioning. However, avoiding traffic loss and guaranteeing that the required QoS is met is not a straightforward task, as it depends on the variance in the traffic flow—defined as the difference between maximum and minimum amount of traffic during some period. Therefore, during this period, the threshold is set conservatively to avoid *underprovisioning* (i.e., traffic exceeds capacity, and some traffic is loss) at the expense of large overprovisioning. (*ii*) Once the variation of the traffic has been determined, a pre-trained *generic model* can be used for flow operation (*Phase II*: time interval [*ta’*, *tb’*]). The model is general as it has been pre-trained assuming a given traffic pattern, e.g., sinusoidal with daily periodicity, but supporting the measured traffic variation. Once in operation, the pre-trained model starts to fine tune with the observed samples. (*iii*) Once enough measurements are available to determine the characteristics of the flow, including the traffic pattern, a specific model can be trained in a sandbox domain by using a simulator set to operate at time *tb’* (*Phase III*). That model should improve the performance or be easier to operate than the pre-trained one. (*iv*) Assuming that the traffic pattern does not change, any change in the traffic variation that cannot be absorbed by the current model can trigger returning to Phase II for more intensive parameter tuning for the new traffic variation, while a new specific model is trained and is set to operate at time *tc’* (Phase II—Phase III cycle).

### 2.2. Proposed Architecture

**Figure 3:**
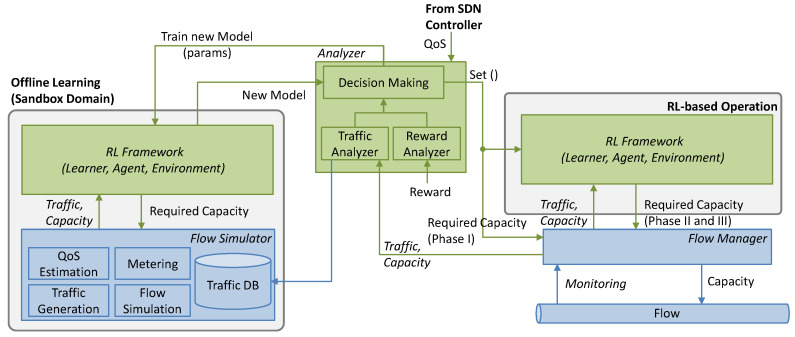
Extended architecture for flow capacity autonomous operation with offline learning.

This work extends the basic RL-based flow capacity operation (Figure 1) and proposes a scheme based on (Figure 3): (*i*) analyzing the traffic to obtain meaningful traffic characteristics; (*ii*) making decisions regarding the allocated capacity when no model is in operation (Phase I); (*iii*) selecting pre-trained models that fit with the observed traffic characteristics; (*iv*) training new models in a sandbox domain (*offline learning*), where real traffic measurements are used to generate traffic in a simulation environment and the QoS can be realistically estimated; a replica of the RL algorithm in operation is used here for training new models; and (*v*) once accurate models are obtained, they are used for flow operation and will be progressively fine-tuned online. As in Figure 1, a *Flow Manager* collects monitoring data from the forwarding plane and enforces flow capacity.

The models include some parameters that need to be tuned as a function of the traffic, to provide the desired performance while meeting the required QoS. Such parameter tuning can be carried out during offline learning, as well as during online operation to deal with small traffic changes. Based on the analysis of the traffic and the reward, the Analyzer block decides when to tune parameters and when to update the model with an offline learned one (labeled *Set*() in Figure 3) to meet the given QoS. Note that both parameter tuning and the offline–online cycle can be completed several times during the operation to improve the learned models, which will also enable adaptability to changes.

The next section details the RL approaches used to solve the autonomous flow capacity management problem, CRUX.

## 3. CRUX Problem Definition and RL Methodology

This section formally defines the CRUX problem, and introduces the main parameters and variables used hereafter. Next, it introduces the methodology to solve the problem using RL, and finally defines the different RL approaches under study. The used notation is summarized in Table 1, where parameters and variables are defined.

### 3.1. Problem Definition and Basic Modeling

Let us consider that the autonomous flow capacity management problem is solved periodically, when a new set of measurements, statistics, and parameters for the traffic flow *x* are collected and computed. Along this section and the following, we adopt the *informational representation* of time, where *t* represents a point in time that refers to the time interval [*t* − 1, *t*) [22]. Specifically, *x*(*t*) represents the traffic measurements collected in [*t* - 1, *t*), and statistics, such as the maximum (*x_max_*(*t*)) and variation (*x_var_*(*t*)), summarize traffic dynamicity during that time interval. Additionally, decisions made at time *t*, e.g., the capacity to be allocated (*z*(*t*)), consider data that arrived up to time *t*.

The main objective of the CRUX problem is to find the optimal (minimum) capacity *z*(*t*) at time *t* that satisfies a desired QoS. In this work, we assume that that the QoS is defined by a desirable maximum end-to-end delay *d_max_*; this also entails that packet loss is not tolerated during flow operation.

**Figure 4:**
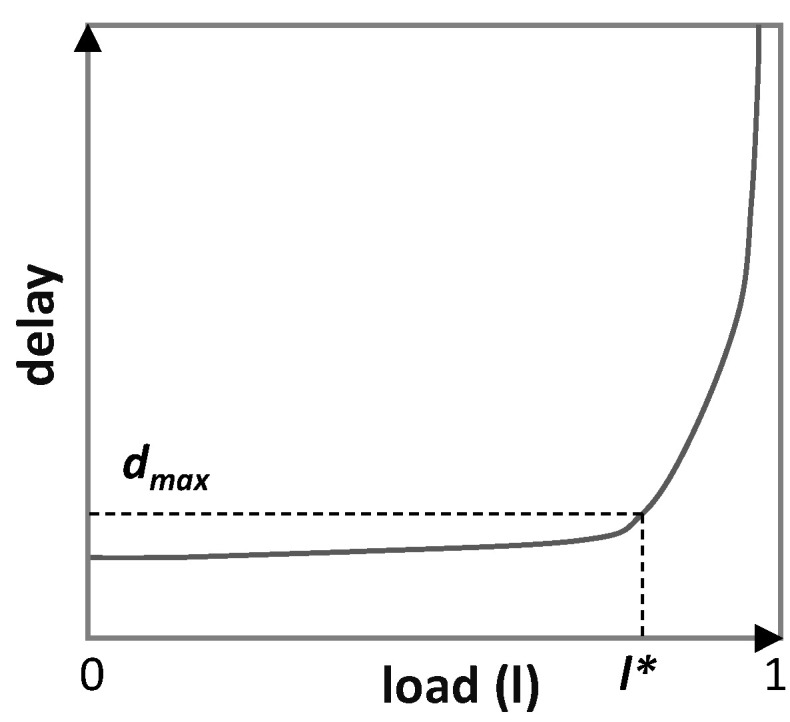
Delay model example.

Without loss of generality, we assume that the delay can be modeled as a function of the traffic volume, the allocated capacity for the flow, the *load* (ratio traffic/capacity), and other components such as the transmission delay (*load–delay* models). Such load–delay models can be obtained during the commissioning testing phase using, e.g., active monitoring techniques [20]. Once the model is available, a target load l* unleashing the target maximum delay *d_max_* can be selected. Figure 4 illustrates an example of a load–delay model, where *d_max_* has been selected to a value where queueing delay becomes the predominant delay component, e.g., for l* = 80%.

A policy (threshold)-based approach can be used to make decisions from the currently available monitoring data, as defined in Equation (1), where *k* is a constant factor that is related to the traffic dynamicity and variability; *k* needs to be tuned to guarantee the required QoS. However, finding the proper value of *k* is not a straightforward task: if the value of *k* is high, QoS is ensured at the cost of high overprovisioning, whereas if the value of *k* is low, QoS requirements might be not met. In addition, decisions are reactive, so altogether, *sub-optimal solutions* are usually obtained.

(1) $$z\left(t\right)=k\cdot x_{max}\left(t\right)/l^{*}$$

The optimal capacity allocation to the CRUX problem requires knowledge of the expected traffic to allocate the capacity of the flow at time *t* − 1 to the value that fits the expected maximum load for the period [*t* − 1, *t*) (proactive decision making):

(2) $$z^{*}\left(t-1\right)=x_{max}\left(t\right)/l^{*}$$

In the case that the capacity allocation is not optimal, some capacity slack/surplus (*o*) will exist, which can be formally computed at time (*t*) as follows:

(3) $$o\left(t\right)=z\left(t-1\right)-x_{max}\left(t\right)/l^{*}$$

**Figure 5:**
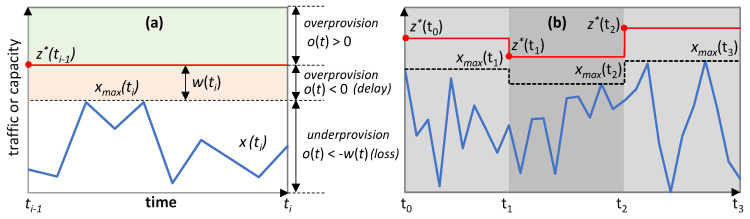
Capacity allocation definition (**a**) and evolution (**b**).

Figure 5 sketches an example of a traffic flow *x*(*t*) for which some capacity allocation *z*(*t*) is required. In the figure, the optimal capacity z*(*t*) that should be allocated at time *t_i_*_−1_ is shown. The different colors provide a visual representation of the values of *o*(*t*). In particular, two different sub-optimal capacity allocations can be distinguished (see labels in Figure 5a): (*i*) if *z*(*t*) > z*(*t*) (i.e., *o*(*t*) > 0), QoS requirements are met at the expense of an excess of overprovisioning; (*ii*) if *z*(*t*) < z*(*t*) (i.e., *o*(*t*) < 0), QoS requirements are violated.

The width *w*(*t*) of the high delay area is formally defined as a function of the maximum traffic in Equation (4). Therefore, traffic loss appears if *o*(*t*) ≤ −*w*(*t*).

(4) $$w\left(t\right)=x_{max}\left(t\right)*\frac{1-l^{*}}{l^{*}}$$

It is worth noting that the quality of a solution taken at time *t* − 1 can only be evaluated at time *t*, which motivates the use of RL to learn the optimal policy that allocates the minimum value of *z*(*t*) to meet the QoS requirements. The details of the RL-based methodology are presented in next subsections.

### 3.2. Generic RL-Based Methodology

**Figure 6:**
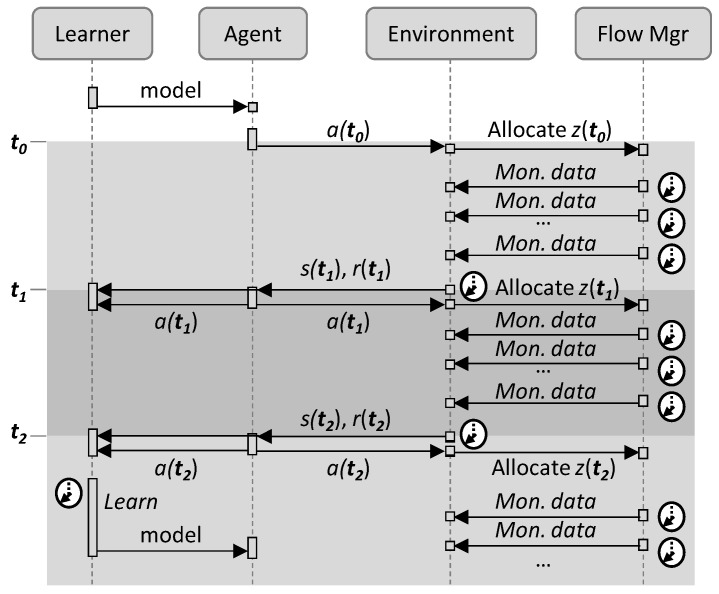
General RL workflow.

Figure 6 illustrates the RL workflow, where the main three elements involved are represented, namely: (*i*) the learner in charge of learning the optimal policy; (*ii*) the agent in charge of taking actions to adjust the capacity allocated to the flow; (*iii*) the environment adaptation module in charge of implementing and evaluating the actions taken; and (*iv*) the flow manager, which enforces the capacity and collects traffic measurements. Three time periods are specified, from *t_0_* to *t_2_*; let us assume that some initial policy model has been set in the agent before operation starts at time *t_0_*, when the agent applies the first action *a*(*t*_0_).

For the sake of simplification and to reduce complexity, action *a*(*t*) is defined in Equation (5) as the differential capacity with regard to the current one. Actions are processed by the environment, which computes the new capacity *z*(*t*) to be allocated.

(5) a(t)=z(t)−z(t−1)

The flow manager periodically sends traffic monitoring data to the environment, which processes them at the end of every time interval to compute state *s*(*t_i_*) and reward *r*(*t_i_*). Upon receiving the state, the agent finds the action *a*(*t_i_*) to be taken with the current policy. In addition, the learning process uses state, reward, and action to improve the model, which is updated in the next time interval. The state function *s*(*t*) is defined in terms of *o*(*t*) normalized by a parameter *y*(*t*) (Equation (6)), which is conveniently set up using parameter *ρ* to absorb the traffic variation observed in the flow (Equation (7)).

(6) s(t)=o(t)/y(t)

(7) $$y\left(t\right)=\rho \;\cdot \;x_{var}\left(t\right)$$

**Figure 7:**
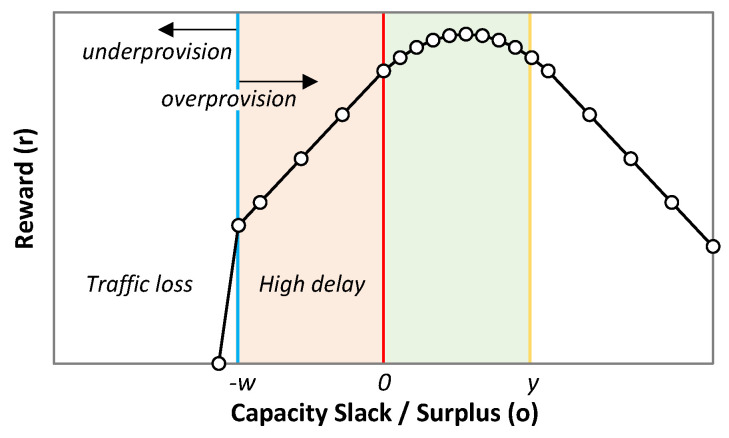
Reward function vs. capacity slack/surplus.

As for the reward *r*(*t*), the objective is to minimize overprovisioning, without providing high delay. To ensure that, the higher delay must be obtained by producing some overprovisioning. To that end, we have defined a piece-wise function with four different regions in the range of *o*(*t*), representing the sub-optimal cases. Such division allows for individual modes of operation that correspond to an adequate reward in each case. The reward function *r*(*t*) is formally expressed in Equation (8) and illustrated in Figure 7. The first and second components of *r*(*t*) penalize traffic loss and high delay, respectively. Both components are linear functions of *o*(*t*), where coefficients *β*_1_ and *β*_2_ can be tuned to penalize traffic loss and high delay. The third component gives the maximum reward, which is slightly shifted to the positive values of *o*(*t*) to reduce the risk of QoS violation. This segment is concave quadratic with regard to the relation *o*(*t*)/*y*(*t*), with the maximum value weighted by coefficient *β*_3_. Finally, overprovisioning above *y*(*t*) is linearly penalized by coefficient *β*_4_.

(8) $$r\left(t\right)=\{\begin{array}{ll} \beta_{1}\cdot \left(o\left(t\right)-w\left(t\right)\right)+\beta_{2}\cdot w\left(t\right), & o\left(t\right)<-w\left(t\right)\\ \beta_{2}\cdot o\left(t\right), & -w\left(t\right)\leq o\left(t\right)<0\;\;\\ \beta_{3}\cdot \left(1-\frac{o\left(t\right)}{y\left(t\right)}\right)\cdot \frac{o\left(t\right)}{y\left(t\right)}, & 0\leq o\left(t\right)<y\left(t\right)\\ -\beta_{4}\cdot \left(o\left(t\right)-y\left(t\right)\right), & o\left(t\right)\geq y\left(t\right) \end{array}$$

### 3.3. Specific Adaption of RL Approaches

As introduced in Section 1, three RL methods are considered to solve the CRUX problem, namely: (*i*) Q-learning, (*ii*) D3QN, and (*iii*) TD3.

For each method, the main adaptation of the generic problem definition is to discretize state and action spaces. Q-learning requires discretizing the continuous state function *s*(*t*) in Equation (6) into a number of discrete states (*n_s_*). Such discrete state function *s’*(*t*) can be formally expressed as Equation (9). Discrete states 0 and *n_s_* − 1 indicate underprovisioning and overprovisioning above margin *y*(*t*), respectively, whereas the rest states *n_s_* − 2 are used to evenly discretize the overprovisioning below margin *y*(*t*).

(9) $$s\prime \left(t\right)=\{\begin{array}{cc} 0, & s\left(t\right)<0\\ (n_{s}-2)\cdot s\left(t\right), & s\left(t\right)\in \left[0,1\right]\\ n_{s}-1, & s\left(t\right)>1 \end{array}$$

In addition, Q-learning and all DQN variants require a discrete space of *n_a_* actions. Let us define *a’*(*t*) as the set of discrete actions, where a discrete action is defined by an integer number of units of capacity *b* to update (add or subtract) the current capacity. The discrete set of actions that depend on both *n_a_* ∊ 2 * ℕ − 1 (natural odd number) and *b* can be formally defined as:

(10) $$a^{\prime }\left(t\right)\in \left\{b\cdot i,\;i\in \left[-\frac{n_{a}-1}{2},\frac{n_{a}-1}{2}\right]\right\}$$

Table 2 summarizes the main characteristics and parameters to be configured for each method.

## 4. Cycles for Robust RL

This section is devoted to the details of the operation lifecycle presented in Figure 2b. Let us assume that when a new path for a flow is set up, an instance of every element in the architecture in Figure 3 is instantiated. The characteristics of the instances depend on the flow requirements, e.g., pre-trained generic models loaded in the *Offline Learning* block are those that were trained with similar QoS requirements (d_max_ and q_a_) to those of the current flow. When the operation starts, no *online RL* models exist and the maximum capacity for the flow *z_max_* is allocated. All the algorithms presented next run in the *Analyzer* block (see Figure 3), which makes decisions and orchestrates the rest of the blocks based on some analysis results.

Algorithm 1 shows the Analyzer initialization that receives the pointers to external modules that interact with the *Analyzer*, i.e., *offline learner, online RL*, and *flow* *manager*, and stores them (line 1 in Algorithm 1), and initializes the main variables used by the rest of the procedures. In particular, *DB* contains the needed traffic-related data for the analysis carried out at every phase (line 2), *params* is a vector with the parameters that characterize flow’s requirements and some configuration and that are used during the different phases (line 3), and *phase* records the current phase, which is initialized to Phase I (line 4).
**Algorithm 1**. Analyzer Initialization**INPUT**: *offlineLearn, onlineRL, flowMgr*  **OUTPUT**: -              1: *store*(*offlineLearn, onlineRL, flowMgr*)
 2: *initialize DB*
 3: *params* ← [<l*, z_max_, k, eps>,   // Phase I  
      <*qa, cfl, ∆ρ*>,      // Phase II  
      <*var_l, var_h, r_l, m>*]  // Phase III  
 4: *phase* ← PhaseI

Before describing the algorithms for the different phases, let us present a specific procedure for traffic variance analysis. Algorithm 2 is applied to the set of observed traffic-related measurements, stored in *DB*, with the objective of characterizing and quantifying the fluctuation of the traffic around its observed average. After retrieving data time series contained in *DB*, the average pattern on the given traffic time series *X* is computed (lines 1–2 in Algorithm 2). Note that the result of this operation produces time series *X_avg_*, with the smoothed average that better fits *X*. Without loss of generality, we assume that a combination of regression techniques including polynomial fitting, spline cubic regression, and sum-of-sin regression is applied, returning the best result in terms of accuracy and model complexity [23]. The relative residuals are computed (line 3) and the difference between maximum and minimum of these residuals (*var*) is considered as the traffic *variance* measurement (line 4). This value together with the previous *var* measurements stored in *DB* (time series *Y*) are used to compute the derivative *drv* of *var* in time, i.e., the first-order difference [24] (lines 5). The last value in *drv* denotes the current derivative, which is later used for the identification of the traffic variance (line 6). In addition, a variance *score* is computed as the maximum absolute derivative value normalized by the observed variance (line 7). The score approaches 1 if the traffic fluctuates around its maximum range between two consecutive time measurements. This score will be used later for generic model tuning purposes. The computed variance results are eventually returned (line 8).
**Algorithm 2.** varianceAnalysis().**INPUT**: *DB***OUTPUT:** *V*
 1: *<X,Y >* ← <*DB.*traffic, *DB*.var>
 2: *X_avg_* ← computeAveragePattern(*X*)
 3: *X_res_* ← (*X*—*X_avg_*) ⨀ *X^−1^*
 4: *var* ← max(*X_res_*)—min(*X_res_*)
 5: *Y*.append(*var*)
 6: *drv* ← computeDerivative(*Y*)
 7: *score* ← max(|*drv|*)/*var*
 8: **return** <var = *var*, curdrv = *drv*[−1], score = *score*>           

Algorithm 3 specifies the main procedure running in the Analyzer block and it is called periodically every time *t*. First, new traffic monitoring data are gathered from the flow manager (line 1 in Algorithm 3). Then, the specific procedure for each phase is called with the current time and the collected data, and the phase changes only when the called procedure returns *True* (lines 2–10); *DB* is initialized every time phase changes (line 11).
**Algorithm 3**. Main Analyzer Procedure**INPUT**: *t***OUTPUT**: -
 1: *x*(*t*)←*flowMgr*.getMonitoringData(*t*)
 2: **if** *phase* = PhaseI **then**
 3:  *changePhase* ← thresholdBased(*t*, *x*(*t*))
 4:  **if**
*changePhase* **then**
*phase* ← PhaseII
 5: **else if** *phase* = PhaseII **then**
 6:  *changePhase* ← modelSelectionAndTuning(*t*, *x*(*t*))         
 7:  **if**
*changePhase* **then**
*phase* ← PhaseIII
 8: **else** // *phase* = PhaseIII
 9:  *changePhase* ← specificModel(*t*, *x*(*t*))
  10:  **if**
*changePhase* **then**
*phase* ← PhaseII
  11: **if**
*changePhase*
**then**
*initialize DB*

Algorithm 4 defines the operation of the *Analyzer* block during Phase I. Recall that during this phase, flow capacity allocation is managed following a threshold-based procedure, defined by Equation (1). First of all, *DB* is updated with the new monitoring data and the variance analysis described in Algorithm 2 is executed, storing the result in variable *V* (lines 1–2 in Algorithm 4). Then, the absolute value of the current derivative is compared with a small epsilon value (param *eps*) to decide whether enough traffic data have been already analyzed to estimate variance with high accuracy (line 3). If so, a generic pre-trained model *f_0_* for the computed variance is retrieved from the *offline learner* and factor *ρ_0_* is scaled with the ratio of scores between the generic model and the observed traffic (lines 4–5); the scaled factor increases (decreases) if the computed score is higher (lower) than the score of the generic model. The rationale behind such factor correction is to achieve a more robust and conservative operation of the generic model under the actual traffic variance behavior. Then, the *online RL* module is updated with new model *f_0_* and scaled factor *ρ_1_* and Phase I ends (lines 6–7).
**Algorithm 4**. thresholdBased() (Phase I)**INPUT**: *t*, *x*(*t*)**OUTPUT:** *changePhase*
 1: *DB.*traffic.append(*x*(*t*))
 2: *V* ←varianceAnalysis(*DB*)
 3: **if** |*V*.curdrv| < *eps*
**then**
 4:    *<f*_0_, *ρ*_0_, *sc*_0_*>*← *offlineLearn*.getGenericModel(*V.*var)       
 5:    *ρ_1_*←*ρ_0_*·(*sc_0_* / *V*.score)
 6:    *onlineRL*.setModel(*f_0_*, *ρ_1_*)
 7:    **return True**
 8: *DB*.var.append(V.*var*)
 9: *k*(*t*)←max(1, *V*.var / (1−l*))
  10: **if** *k*(*t*)>*k* **then**
*k*←*k*(*t*) **else**
*k*←*k* − (*k*(t) − *k*)/2
  11: *flowMgr*.setupCapacity(max(*k ·* max(*x*)/l*, z_max_))
  12: **return False**

In case the current derivative is still high, the threshold-based capacity allocation procedure continues. Here, factor *k* in Equation (1) is adapted from its input value as soon as more traffic data are available and traffic variance is better estimated. With the estimated variance and target load l*, factor *k*(*t*) is computed. Then, *k* is updated in two different ways: (*i*) reducing by half between *k* and *k*(*t*) if *k* is larger than needed; or (*ii*) replaced by *k*(*t*) if is *k* is lower than needed (lines 8–10). The flow manager is requested to modify the flow capacity to the computed *z*(*t*), which is bounded by the maximum capacity *z_max_* (line 11) and Phase I continues (line 12).

Algorithm 5 details the procedure during Phase II. Traffic data collected from the *flow manager* are sent to the *offline learner* block for updating a historical traffic database used to train RL models offline in the sandbox (line 1 in Algorithm 5). The offline model training procedure runs in parallel in the sandbox domain. When enough traffic measurements are collected and processed and an accurate and robust offline-trained RL model is available, it is sent to the *online RL* block to be used for flow capacity operation; this ends Phase II (lines 2–5). Otherwise, Phase II continues, aiming to identify whether the RL model currently in operation needs some parameter tuning (in addition to model updates that the RL algorithm performs during operation). In particular, this procedure aims to supervise the degree of QoS assurance (as compared with the target value, *qa*) obtained by the current model and modifying factor *ρ* when needed to achieve the target performance. Note that low overprovisioning is the secondary objective and therefore, QoS assurance analysis requires computing whether the current capacity violated maximum delay (*o*(*t*) < 0) or not (*o*(*t*) ≥ 0). The result is stored in *DB* (lines 6–10). Next, two different one-sample proportion binomial hypothesis tests [25] are conducted to detect whether the observed degree of QoS assurance is significantly below (test 1) or above (test 2) the target value *qa* (lines 11–13). In the case that some of the hypotheses can be confirmed (on the contrary, it is assumed that QoS assurance is in the target), *ρ* needs to be tuned. If hypothesis test 1 is confirmed, some extra capacity allocation is needed, which is achieved by increasing *ρ* with a given step size *∆ρ*. On the contrary, if hypothesis test 2 is confirmed, allocated capacity can be reduced, so *ρ* is decreased by the same step size.
**Algorithm 5**. ModelSelectionAndTuning() (Phase II)**INPUT**: *t*, *x*(*t*)**OUTPUT:** *changePhase*
 1: *offlrn*.updateTrafficDB(*x*(*t*))
 2: **if** *offlineLearn*.newModelAvailable() **then**
 3:  *<f*, *ρ>*← *offlineLearn*.getModel()
 4:  *onlineRL*.setModel(*f*, *ρ*)
 5:  **return True**
 6: *x_max_*(*t*) = max(*x*)
 7: *z*(*t* − 1)← *flowMgr*.getCurrentCapacity(*t*)
 8: *o*(*t*)←computeSlackSurplus(*x_max_*(*t*), *z*(*t* − 1))//Equation (3)       
 9: **if**
*o*(*t*)<0 **then** *DB*.QA.append(0)
  10: **else** *DB*.QA.append(1)
  11: *p_obs_*←avg(DB.QA)
  12: *pval_l*←BinomialTest1(“*p_obs_* < *qa*”)
  13: *pval_g*←BinomialTest2(“*p_obs_* > *qa*”)
  14: **if** min(*pval_g*, *pval_l*) > *cfl* **then**
  15:  *DB*.QA←∅
  16:  **if**
*pval_l* < *cfl*
**then**
*onlineRL.*tuneParam(‘*ρ*’, ∆*ρ*)
  17:  **else**
*onlineRL.*tuneParam(‘*ρ*’, −∆*ρ*)
  18: **return**
**False**

Finally, Algorithm 6 describes the procedure running in the Analyzer during Phase III. This algorithm analyzes the last m traffic measurements and the reward obtained by the *online RL* (lines 1–6 in Algorithm 6). The objective of this analysis is to check whether both the current traffic and reward follow the expected behavior (line 7). Let us assume that an extended estimation of the working variance with range [*var_l*, *var_h*] is found during the offline training phase—with a minimum and maximum variance that the RL model can support without losing either robustness or desired performance. Bear in mind that operating a traffic flow with more variance than what is supported by the model can lead to poor QoS assurance and even traffic loss. On the contrary, a traffic flow with less variance can produce large overprovisioning, which the *online RL* can hardly decrease with its fine adaption configuration. In fact, *online RL* continuously adapts the model to smooth traffic changes with controlled reward fluctuations, so that a minimum reward (*rw_l*) can be considered as the reasonable limit of a normal RL operation. Therefore, Phase II is triggered back when traffic variance leaves the working range of the RL model or the observed reward goes below that limit; otherwise, Phase III continues.
**Algorithm 6**. specificModel() (Phase III)**INPUT**: *t*, *x*(*t*)**OUTPUT:** *changePhase*
 1: *rw*(*t*) ← *onlineRL*.getReward(*t*)
 2: *DB.*traffic.append(*x*(*t*))
 3: *DB*.reward.append(*r*(*t*))
 4: **if**
*|DB*.traffic*| > m* **then** *DB*.traffic.pop(0)
 5: **if**
*|DB*.reward*| > m* **then** *DB*.reward.pop(0)
 6: *V*←varianceAnalysis(*DB*)
 7: **return**
*V.*var NOT IN [*var_l*, *var_h*] OR *rw*(*t*) < *rw_l*        

## 5. Illustrative Results

For the ongoing evaluation, a Python-based simulator reproducing the modules described in Figure 3 was implemented. Realistic traffic flow behavior was accurately emulated using a simulator based on CURSA-SQ [26]. CURSA-SQ combines statistically based traffic flow generation and continuous G/G/1/k queue model based on the logistic function; multiple queuing systems can be numerically analyzed and related statistics, such as traffic magnitude, queuing delay, and packet loss, be computed. CURSA-SQ was validated with the experimental measurements in [20] and was used to successfully reproduce realistic packet scenarios [11,27]. In the context of this work, CURSA-SQ is used as: (*i*) a lightweight network simulator to emulate a flow manager and the forwarding plane; and (*ii*) a flow simulator running in the ML sandbox domain for offline RL training purposes. It is worth highlighting that both CURSA-SQ instances have been independently configured and managed in order to reproduce the actual separation between the physical network and the sandbox domain.

Traffic was randomly generated according to different *traffic configurations*. Each traffic configuration is the combination of traffic pattern and variance. Two different daily patterns were considered: a simple *sinusoidal* pattern for offline training purposes, and a *realistic* pattern [16] to emulate the real traffic in the forwarding plane. In both cases, traffic fluctuates between 5 Gb/s (valley) and 40 Gb/s (peak) throughout the day. Regarding variance, it is defined as a percentage of the mean, so the magnitude of traffic oscillations changes in time (heteroscedasticity). For the sake of a wider analysis, we considered five different variance values: 1%, 3%, 6%, 12%, and 25%. 

RL algorithms running in the RL-based operation and offline learning modules have been implemented in Python3 using libraries such as *pytorch*. A general epsilon decay strategy was implemented in all the RL methods for balancing between exploration and exploitation [7], with decay factor equal to 0.00125. Moreover, a discount factor equal to 0.95 was set up. Q-learning was configured with *n_s_* = 14 states and *n_a_* = 3 actions, as well as capacity allocation granularity *b* = 1 Gb/s. In the case of D3QN and TD3, every DNN consisted of two hidden layers with 100 neurons each implementing the Rectified Linear Unit activation function. All DNNs were trained by means of the Adam replacement optimizer with learning rate equal to 0.001 and maximum replay buffer equal to 1e6 samples.

Finally, the capacity and QoS parameters for the flow under study are maximum capacity *z_max_* = 100 Gb/s, optimal load l* = 80%, and QoS assurance *qa* = 99%.

In the next two subsections, we first focus on comparing the different RL methods for the scenario where the pure online learning RL-based operation is performed (see Figure 2a). Next, we evaluate the offline leaning + online RL-based operation (Figure 2b), including the three proposed phases.

### 5.1. Online RL-Based Operation

**Figure 8:**
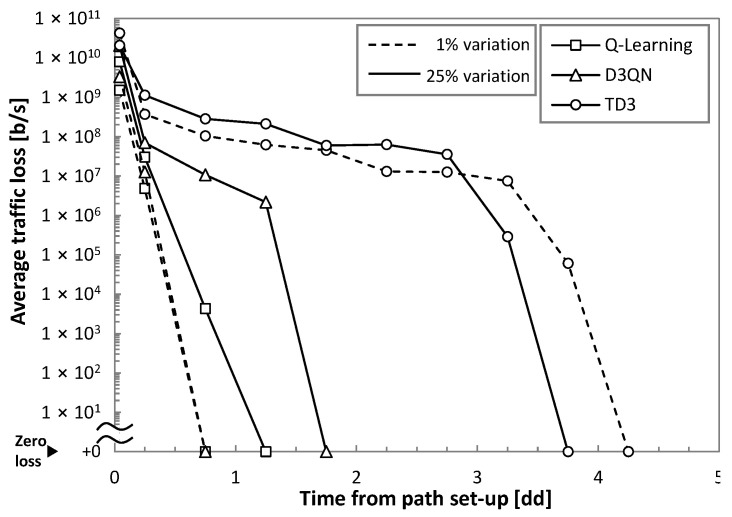
Achieving zero loss operation.

Let us first analyze the reliability of the RL operation under real traffic; we focus specifically on the traffic loss. For this study, low (1%) and high (25%) variances were considered. Figure 8 plots the traffic loss as a function of time from the path set-up time. We observe extremely poor performance (high loss) at the beginning of operation, as it was anticipated in Figure 2a. Interestingly, we observe that the simplest Q-learning method provides the fastest convergence time to achieve zero loss, although it needs more than one day to achieve zero loss operation when traffic variance is high. Note that D3QN is the most sensitive to traffic configuration (zero loss operation time increases three times from low to high variance). TD3 is the method with the slowest convergence (around 4 days).

**Figure 9:**
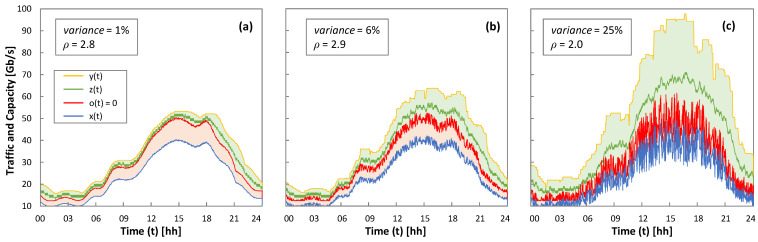
Q-Learning operation. Traffic and allocated capacity for low (**a**), moderated (**b**), and high (**c**) traffic variance.

As all the RL methods have achieved zero loss operation, the rest of the results analyze the performance after 5 days of operation. As an illustrative example of the RL-based operation, Figure 9 shows one day of real traffic *x*(*t*) and variance from low to high, as well as the capacity *z*(*t*) allocated using Q-learning; optimal *ρ* for each variance is configured. The optimal capacity allocation (*o*(*t*) = 0) and the margin for overprovisioning *y*(*t*) are also plotted. We observe that the allocated capacity is close to the optimal one, absorbing fluctuations with enough margin to meet the target QoS.

**Figure 10:**
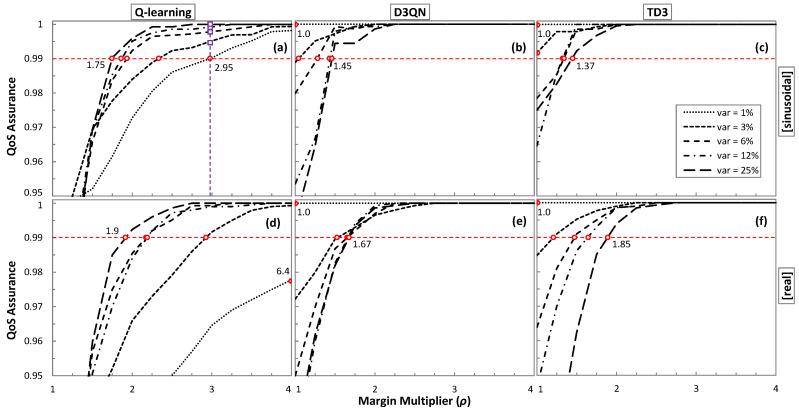
QoS as a function of ρ models trained with a sinusoidal traffic pattern (**a**–**c**) and real traffic (**d**–**f**).

Let us now analyze the impact of the margin multiplier *ρ* to achieve the desired QoS. Figure 10 shows the obtained QoS assurance as a function of *ρ*. For the sake of a comprehensive study, all traffic configurations for sinusoidal (Figure 10a–c) and real (Figure 10d–f) traffic patterns have been analyzed. The minimum *ρ* value has been set to 1. The *ρ* values for which the target QoS of 99% is achieved are highlighted with a round marker. We observe in the results that *ρ* depends not only on the traffic characteristics, but also on the RL method.

Interestingly, Q-learning needs the widest range of values for all traffic configurations, requiring a smaller *ρ* as soon as variation increases. It is worth noting that the large range of values ([1.9, 6.4]) makes it more difficult to adjust *ρ* for different traffic configurations. Conversely, D3QN and TD3 show a smaller *ρ* range and opposite behavior as *ρ* increases with the traffic variance.

**Figure 11:**
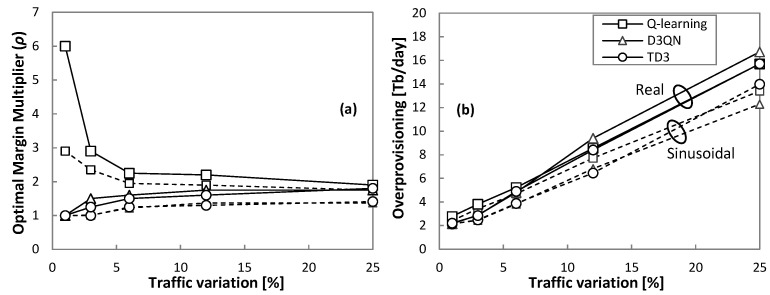
Optimal margin multiplier (**a**) and overprovisioning (**b**).

**Figure 12:**
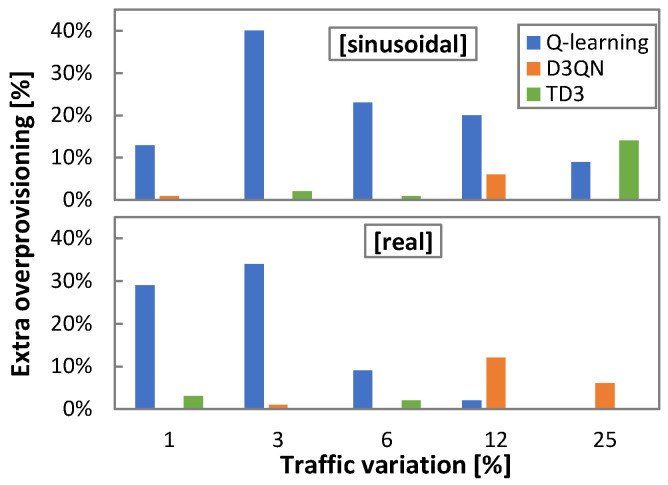
Relative extra overprovisioning.

The detailed evolution of the optimal *ρ* with respect to the traffic variation is plotted in Figure 11a, and Figure 11b shows the total overprovisioning introduced by every RL method operating with the optimal *ρ*. We observe that overprovisioning increases with traffic variation, with a slightly different trend depending on the traffic pattern (sinusoidal and real). Moreover, every RL method introduces different amounts of overprovisioning as shown in Figure 12, where the relative overprovisioning per RL method with respect to the minimum one for every traffic configuration is represented. Q-learning is the method that requires larger overprovisioning in general terms, whereas D3QN and TD3 show better performance. Interestingly, the differences are proportionally larger when traffic variation is small.

Let us analyze the effect in terms of extra-overprovisioning when *ρ* is fixed to a constant value, high enough to assure reliable QoS performance in online RL operation under traffics with a wide range of characteristics. The squared purple markers in Figure 10a indicate the QoS that could be achieved under different traffic characteristics for the largest *ρ*. Table 3 summarizes the relative and absolute increment of overprovisioning (computed in total Tb per day of operation) produced with the most conservative *ρ* configuration for all traffic configurations and RL methods. When the fixed *ρ* happens to be the optimal one for the traffic and RL method, no additional overprovisioning is set up (values in boldface); otherwise, additional overprovisioning is introduced. Q-learning is the method that adds the largest extra overprovisioning (exceeding 200% and 30 Tb/day). In any case, it is worth highlighting that achieving optimal performance in terms of QoS assurance while achieving efficient capacity allocation requires some method to find the optimal *ρ* for the traffic characteristics.

To conclude this section, we can highlight (*i*) that the CRUX problem proposed in Section 2 can be tackled using RL; under different traffic characteristics and RL methods, the target QoS is assured with reduced overprovisioning; (*ii*) online RL operation leads to traffic loss at the beginning of flow capacity operation; this fact prevents us from using RL until online learning has come up with a robust and reliable model to guarantee autonomous flow operation; and (*iii*) the comparison of the RL methods shows interesting differences among them. While Q-learning learns fast, it also produces larger overprovisioning. D3QN and TD3 need more time to ensure zero loss and adjust QoS at the benefit of reducing overprovisioning.

### 5.2. Offline Leaning + Online RL-Based Operation

Let us now focus on evaluating the operational approach detailed in Section 4 (Figure 2b), which consists of three phases. Before emulating flow operation, we generated synthetic data for all the traffic variances following the sinusoidal traffic pattern and used them to pre-train generic models independently for each traffic configuration and RL method. We ran every offline RL training for 14,400 episodes to guarantee QoS assurance with minimum overprovisioning.

**Figure 13:**
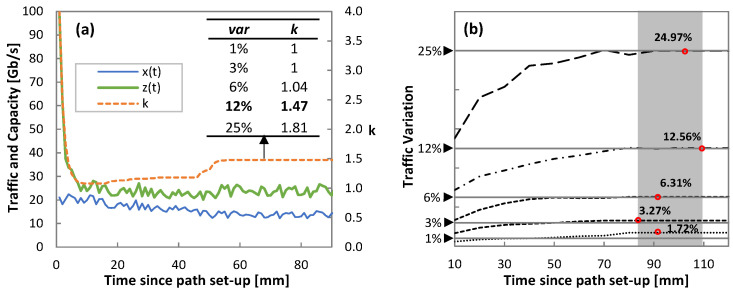
Phase I: Self-tuned threshold (**a**) and traffic variance analysis (**b**).

Starting with the analysis of Phase I, Figure 13a shows an illustrative example of the evolution of the capacity during the operation of the threshold-based algorithm (Algorithm 4) for the real traffic pattern and variation 12%. Actual traffic *x*(*t*), allocated capacity *z*(*t*), and the evolution of the self-tuned *k* parameter are also shown. Note that *k* quickly evolves from its initial value (*k* = 4) to reach a capacity closer to actual traffic. However, as soon as the load exceeds the optimal one l*, *k* is increased until reaching a stable value (1.47), which happens after 60 minutes of operation. The inset table in Figure 13a details the values of *k* after one hour of operation for all traffic configurations. It is worth noting that the self-tuned threshold-based algorithm operates with zero traffic loss for all the cases.

Parallel to the threshold-based operation, traffic variance analysis (Algorithm 2) is conducted in order to compute the true variance of the traffic. Figure 13b shows the computed traffic variation as a function of time for all traffic configurations. Round markers highlight when the derivative of traffic variance reached a small *eps* = 0.01 and labels show the final computed variance value. The low error between computed and true variances is noticeable. Such estimation is achieved within two hours in all the cases.

**Figure 14:**
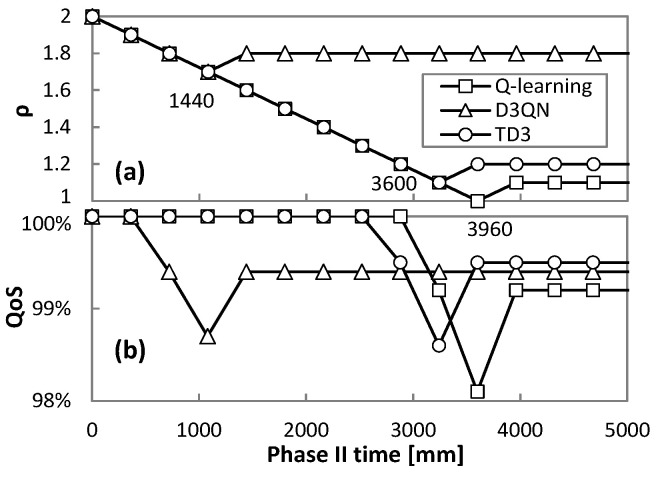
Phase II: QoS (**a**) and ρ (**b**) evolution.

After two hours of operation, *Phase II* (Algorithm 5) can start and an RL method with a generic pre-trained model for the true traffic variance is set to operate. The tuning of parameter *ρ* (∆*ρ* = 0.1) and the resulting QoS are shown in Figure 14a,b, respectively, for traffic variance equal to 12%. To detect whether the measured QoS is considerably below or above the desired *qa* value, a significance level *cfl* = 0.05 was used to be compared against the obtained *p*-value from the binomial tests. We observe that *ρ* decreases up to a magnitude that produces QoS below 99%; just after that, *ρ* increases and remains stable from that point on. As shown in Figure 14a, the time to converge to the best *ρ* is 3960, 1440, and 3600 min (2.75, 1, and 2.5 days) for Q-learning, D3QN, and TD3, respectively.

**Figure 15:**
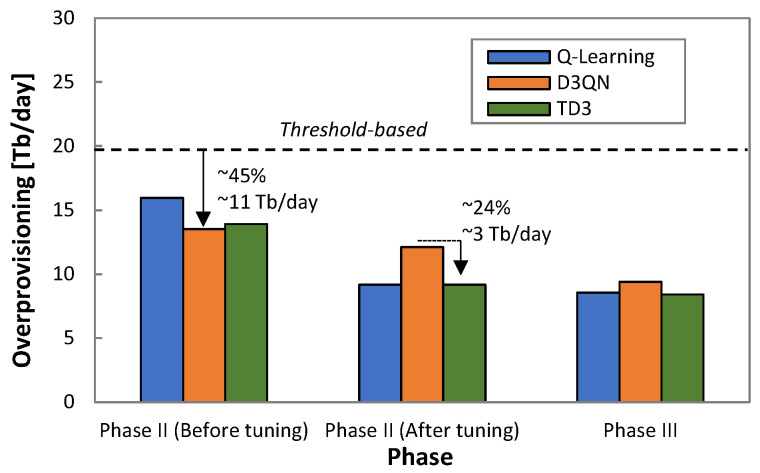
Overprovisioning reduction.

The above analysis, however, needs to be complemented with the overprovisioning to extract meaningful conclusions. Figure 15 presents the overprovisioning obtained by every RL method before and after tuning *ρ* in *Phase II*. For reference purposes, the overprovisioning introduced by the threshold-based algorithm during *Phase I* is also included as a dotted line. The large benefits in terms of overprovisioning reduction for the RL-based operation with regard to the threshold-based algorithm are remarkable—up to 45% of capacity allocation reduction and 11 Tb/day of total capacity savings for one single flow. After *ρ* tuning, D3QN shows the worst performance, as Q-learning and TD3 achieved significantly lower overprovisioning (24%, ~3 Tb/day). Figure 15 also shows the obtained overprovisioning when the specific model (trained offline with the collected traffic) is loaded in *Phase III* after 10 days of operation. We observe that Q-learning and TD3 reduce overprovisioning slightly, whereas a larger reduction is achieved with D3QN; we conclude that the former RL methods are less dependent on an accurate model of the specific traffic to achieve optimal capacity allocation.

**Figure 16:**
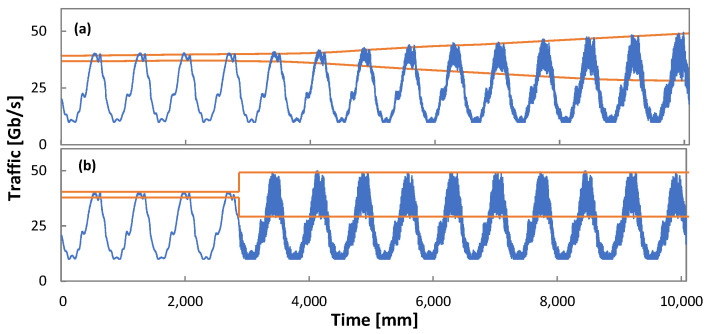
Phase III: Traffic variance change scenarios. Gradual increase (**a**) and sudden increase (**b**).

Finally, let us analyze the performance of Algorithm 6 to detect traffic changes while flow is operated in *Phase III*; recall that such detection immediately triggers *Phase II.* To this end, we generated four different scenarios, combining two different types of changes in traffic variance while keeping traffic profile unchanged. We evaluate *gradual* and *sudden/increase* or *decrease* traffic variance changes. Figure 16 illustrates two out of four scenarios: *gradual increase* (variance gradually increases from 1% to 25% along 5 days) and *sudden increasing* (from 1% to 25% in just one minute); an inverse trend is configured for *gradual* and *sudden decrease* scenarios. 

Table 4 details the time to detect the traffic change when the variance range was configured as [*var_l*, *var_h*] = [−10%, +10%]; the current traffic variance and minimum reward *rw_l* was set to 5% of the minimum observed reward (see Algorithm 6). We observe that the proposed mechanism ensures prompt reaction under any of the studied changes—immediate detection is achieved when a sudden change happens, and no more than one hour is required for gradual change detection.

To evaluate the promptness of detection, Table 4 considers the observed QoS at the detection time, as well as the elapsed time between detection time and the time when reward begins to degrade (reveals whether the RL module is working properly). Note that the detection happens when the QoS is still above the target value in all the cases. This is proof of anticipation of the change detection, which is key to guarantee robust and reliable RL-based operation.

## 6. Concluding Remarks

The Flow Capacity Autonomous Operation (CRUX) problem has been introduced to deal with online capacity allocation of traffic flows subject to dynamic traffic changes; it guarantees precise QoS requirement assurance and minimizes capacity overprovisioning. RL-based operation was proposed to learn the best policy for the traffic characteristics and QoS requirements of a given flow. RL allows adaptive and proactive capacity allocation once the optimal policy is learnt. However, pure RL operation lacks robustness during online learning (e.g., at the beginning of flow operation and in the event of traffic changes) and might result in undesirable traffic loss. However, this can be avoided using simpler reactive threshold-based capacity allocation.

In view of the above, an offline + online learning lifecycle was proposed, aiming at providing guaranteed performance during the entire lifetime of the traffic flow. The proposed management lifecycle consists of three phases. Firstly, a self-tuned threshold-based approach was proposed to operate just after the flow is set up and until enough evidence of the traffic characteristics are available (*Phase I*). Secondly, an RL operation based on models with a pre-trained generic traffic profile but meeting specific traffic variance that was measured during Phase I was executed (*Phase II*). Lastly, an RL operation with models trained for the real measured traffic, while allowing an online RL to adapt to smooth traffic changes (*Phase III*). In addition, during *Phase III* online traffic analysis and RL performance tracking was conducted to detect sharper traffic changes that might require moving back to *Phase II* to keep high reliability.

The proposed lifecycle was implemented under three different RL models, namely, Q-learning, D3QN, and TD3. While Q-learning allows for simple and easy-to-learn definition of policies under discrete spaces of states and actions, D3QN and TD3 enable the application of more complex policies based on deep learning models with continuous state space (D3QN) and continuous action space (TD3).

Numerical evaluation of the proposed offline + online lifecycle under different RL techniques was carried out, reproducing realistic traffic flows in a simulation environment. For benchmarking purposes, comparative results against basic threshold-based operation and online RL operation were also presented. The main conclusions extracted from the numerical evaluation are summarized in Table 5, where colors are used to highlight the results. As expected, online RL produces moderate to high loss (reaching peaks of 1–10 Gb/s) at the beginning of the network operation. Among the different methods, Q-learning reached the required QoS operation earlier (up to 6 times faster than TD3) at the expense of moderate to large overprovisioning (up to 40% larger than TD3). On the other hand, D3QN and TD3 needed more time to converge to the required QoS operation but resulted in considerably better capacity allocation efficiency.

The analysis of the numerical results of the proposed lifecycle leads to several conclusions. Firstly, zero traffic loss and QoS assurance is guaranteed from path set-up regardless of the chosen RL method. Secondly, *Phase II* allows a very efficient and robust operation based on pre-trained generic models that were tuned with specific traffic characteristics. *Phase II* clearly outperformed threshold-based operation in terms of capacity utilization since it remarkably reduced overprovisioning (up to 45%). Thirdly, all the methods reached outstanding capacity efficiency (more than 50% of capacity reduction with respect to threshold-based operation) without losing QoS performance in *Phase III*; Q-learning and TD3 behaved slightly better than D3QN. Finally, the continuous analysis and tracking conducted during *Phase III* to detect traffic changes allowed a prompt detection of sharp changes (between 1 and 650 minutes), triggering *Phase II* from several hours to days before online RL operation suffered any significant degradation.

## Figures and Tables

**Table 1:** Notation.

**Capacity and QoS Params for the Flow**	
*z_max_*	Maximum capacity (Gb/s)
*d_max_*	Target maximum delay (s)
l*	Optimal load (unleashing *d_max_*) ∊ [0, 100]%
*qa*	QoS Assurance (%)
**Traffic and Capacity**	
*x_max_*(*t*)	Maximum traffic at time *t* (Gb/s)
*x_var_*(*t*)	Traffic variation at time *t* (Gb/s)
*z*(*t*)	Capacity allocated at time *t* (Gb/s)
o(*t*)	Capacity slack/surplus at time *t* (Gb/s)
*y*(*t*)	Overprovisioning margin (Gb/s)
*ρ*	Traffic variance multiplier
*b*	Granularity of capacity allocation (Gb/s)
*w*(*t*)	Traffic loss margin (Gb/s)
**Autonomous Capacity Allocation**	
*a*(*t*)	Action time *t* (b/s)
*n_a_*	Number of discrete actions
*s*(*t*)	State at time *t*
*n_s_*	Number of discrete states
*r*(*t*)	Reward at time *t*
*k*	Threshold-based scaling factor
*β_i_*	Reward function coefficients (≥0)

**Table 2:** Summary of RL approaches.

Approach	State Space	Action Space	Parameters
Q-learning	Discrete, Equation (9)	Discrete. Equation (10)	*n_s_*, *n_a_*, *b*
D3QN	Continuous, Equation (6)	Discrete. Equation (10)	*n_a_*, *b,* DNN config, Replay buffer
TD3	Continuous, Equation (6)	Continuous, Equation (10)	Actor/critic DNN config Replay buffer

**Table 3:** Additional overprovisioning when fixing *ρ* conservatively.

Traffic Pattern	Traffic Variance	Q-Learning		D3QN		TD3	
		%	Tb/Day	%	Tb/Day	%	Tb/Day
**Sinusoidal**	1%	0.00%	0	0.88%	0.02	2.49%	0.05
	3%	19.18%	0.67	6.04%	0.16	20.67%	0.51
	6%	38.71%	1.85	5.21%	0.21	15.38%	0.63
	12%	44.15%	3.56	0.00%	0.00	4.01%	0.32
	25%	60.40%	8.29	0.00%	0.00	0.00%	0.00
**Real**	1%	0.00%	0	0.05%	0.00	4.95%	0.11
	3%	106.68%	3.94	9.52%	0.28	0.39%	0.01
	6%	192.50%	9.52	12.72%	0.60	2.90%	0.14
	12%	200.69%	18.37	0.00%	0.00	0.00%	0.00
	25%	219.35%	34.46	0.00%	0.00	0.00%	0.00

**Table 4:** Phase III: Analysis under traffic changes.

Traffic Change Scenario	Detection Time (min)	QoS at Detection Time (%)			Reward Degradation (Min from Detection)		
		Q-L	D3QN	TD3	Q-L	D3QN	TD3
**Gradual increase**	45	99.30	100	100	419	595	585
**Gradual decrease**	650	99.86	99.44	99.72	3354	2440	2233
**Step increase**	1	99.17	99.86	99.86	332	413	433
**Step decrease**	1	99.03	99.44	99.44	494	683	212

**Table 5:** Summary of results for policy-based and RL operation with and without offline learning.

Approach	Concept		Threshold-Based	Q-Learning	D3QN	TD3
Policy-based	Traffic loss		Zero traffic loss			
	QoS assurance		Since path set-up			
	Over-provisioning		Very large			
Online RL Operation	Traffic loss			Moderated loss	Moderated loss	High loss
	QoS assurance			After 2 days	After 2 days	After 5 days
	*ρ* range for QoS assurance			Wide	Narrow	Narrow
	Over-provisioning	conservative *ρ*		Large	Small	Small
		optimal *ρ*		Moderate	Small	Small
Offline + Online RL Operation	Traffic loss		Zero traffic loss	Zero traffic loss	Zero traffic loss	Zero traffic loss
	QoS assurance		Since path set-up	Since path set-up	Since path set-up	Since path set-up
	*ρ* fine tuning effectiveness			Large	Moderated	Large
	Over-provisioning Gain	Phase I	None			
		Phase I → Phase II		Moderated	Large	Large
		Phase II		Large	Small	Large
		Phase II → Phase III		Small	Large	Small
	Reliability (Phase III → Phase II)			High	High	High

## Data Availability

The data presented in this study are available on request from the corresponding author.
